# Traumatic rib fracture patterns associated with bone mineral density statuses derived from CT images

**DOI:** 10.3389/fendo.2023.1304219

**Published:** 2023-12-12

**Authors:** Yilin Tang, Wei Hong, Xinxin Xu, Ming Li, Liang Jin

**Affiliations:** ^1^Radiology Department, Huadong Hospital, Affiliated with Fudan University, Shanghai, China; ^2^Department of Geriatrics and Gerontology, Huadong Hospital, Affiliated with Fudan University, Shanghai, China; ^3^Clinical Research Center for Geriatric Medicine, Huadong Hospital, Affiliated with Fudan University, Shanghai, China; ^4^Diagnosis and Treatment Center of Small Lung Nodules, Huadong Hospital, Affiliated with Fudan University, Shanghai, China; ^5^Radiology Department, Huashan Hospital Affiliated with Fudan University, Shanghai, China

**Keywords:** artificial intelligence, bone mineral density, computed x-ray tomography, fracture risk assessment, rib fractures

## Abstract

**Background:**

The impact of decreased bone mineral density (BMD) on traumatic rib fractures remains unknown. We combined computed tomography (CT) and artificial intelligence (AI) to measure BMD and explore its impact on traumatic rib fractures and their patterns.

**Methods:**

The retrospective cohort comprised patients who visited our hospital from 2017–2018; the prospective cohort (control group) was consecutively recruited from the same hospital from February–June 2023. All patients had blunt chest trauma and underwent CT. Volumetric BMD of L1 vertebra was measured by using an AI software. Analyses were done by using BMD categorized as osteoporosis (<80 mg/cm3), osteopenia (80–120 mg/cm3), or normal (>120 mg/cm3). Pearson’s χ2, Fisher’s exact, or Kruskal–Wallis tests and Bonferroni correction were used for comparisons. Negative binomial, and logistic regression analyses were used to assess the associations and impacts of BMD status. Sensitivity analyses were also performed.

**Findings:**

The retrospective cohort included 2,076 eligible patients, of whom 954 (46%) had normal BMD, 806 (38.8%) had osteopenia, and 316 (15.2%) had osteoporosis. After sex- and age-adjustment, osteoporosis was significantly associated with higher rib fracture rates, and a higher likelihood of fractures in ribs 4–7. Furthermore, both the osteopenia and osteoporosis groups demonstrated a significantly higher number of fractured ribs and fracture sites on ribs, with a higher likelihood of fractures in ribs 1–3, as well as flail chest. The prospective cohort included 205 eligible patients, of whom 92 (44.9%) had normal BMD, 74 (36.1%) had osteopenia, and 39 (19.0%) had osteoporosis. The findings observed within this cohort were in concurrence with those in the retrospective cohort.

**Interpretation:**

Traumatic rib fractures are associated with decreased BMD. CT-AI can help to identify individuals who have decreased BMD and a greater rib fracture rate, along with their fracture patterns.

## Introduction

Blunt chest trauma is a significant global health concern, comprising 15% of emergency department admissions and ranking as the third leading cause of trauma-related deaths, with substantial morbidity and mortality rates (1, 2). It is commonly caused by incidents such as traffic accidents, violent acts, and falls, which are not uncommon in daily life and work, implying that any individual engaged in social activities is at risk of experiencing blunt chest trauma (3, 4). Among these causes, traffic accidents are the most prevalent reason for blunt chest trauma (3). In China, the number of motor vehicles has grown by 250% in the past decade, reaching 319 million by the end of 2022, making it the world’s largest automobile market. This explosive growth and the substantial overall volume have contributed to a consistently high number of blunt chest trauma patients (5).

Rib fractures are the most common associated injuries among these patients and serve as a crucial indicator of injury severity (6). The severity of trauma is influenced by different types of rib fractures. The number of rib fractures is a known risk factor for rib fracture-associated mortality, incidence, intensive care unit admission rate, hospitalization duration, and lung injury (7, 8). In some countries, the number of rib fractures is also a forensic assessment indicator for accident penalties and sentencing (9). Specific rib fractures correlate with different organ injuries; ribs 1-3 are associated with a higher risk of major vascular and brachial plexus injuries, ribs 4-7 with an increased risk of lung contusion and cardiovascular injuries, and ribs 8-12 with a higher risk of abdominal solid organ injuries (10). Flail chest, the most severe pattern of rib fractures, necessitates an immediate assessment of the patient’s condition and appropriate measures (11). Therefore, different types of rib fractures have a significant impact on the management and prognosis of patients with thoracic trauma.

Bone mineral density (BMD) is a crucial factor influencing the risk of fracture (12–15). In addition to increasing the susceptibility to fragility fractures, decreased BMD also raise the likelihood of high-energy traumatic fractures (16, 17). With the global aging trend intensifying, the population with decreased BMD is expected to increase, thereby drawing increasing attention to its adverse consequences. However, there is a relative dearth of research regarding how BMD impacts the risk and patterns of rib fractures in patients with blunt chest trauma.

Early identification of osteoporosis/osteopenia is crucial in preventing fractures and reducing associated medical costs (18). Recently, there has been growing interest in the concept of opportunistic BMD screening, which involves extracting BMD information from CT scans performed initially for non-BMD related clinical purposes (19, 20). Additionally, the integration of artificial intelligence (AI) has facilitated the automation of this process, eliminating the laborious manual operations (21, 22). This method, requiring no additional time costs or radiation exposure, provides additional information through a single scan, aiding in the identification of individuals at higher risk of future fractures and their potential patterns of rib fractures. This may contribute to raising awareness in patients, prompting thoughtful consideration of improving BMD and preventing rib fractures. Therefore, this study aims to evaluate the impact of BMD on the risk and patterns of rib fractures following blunt chest trauma through opportunistic BMD screening based on CT-AI.

## Patients and methods

### Study population

**Figure 1:**
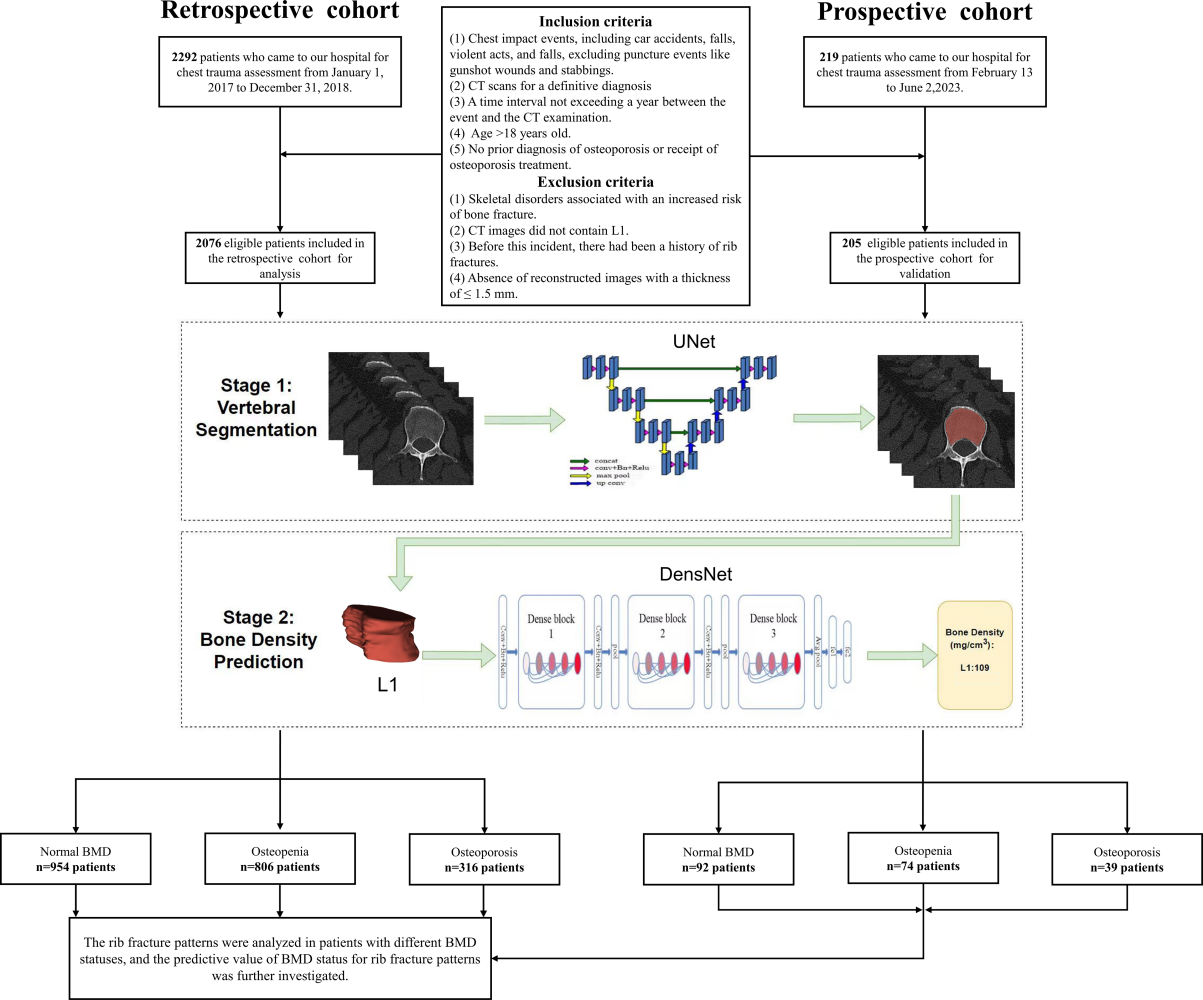
Workflow of this study. CT, computed tomography; BMD, bone mineral density.

This study was approved by the Ethics Committee of Huadong hospital (approval number: 20220069). The requirement to obtain written informed consent was waived. The study dataset of CT images was identified by searching the hospital’s Picture Archiving and Communication System using the keyword “chest trauma evaluation”. The inclusion criteria included: (1) individuals who had experienced chest collision events, such as car accidents, violent acts, and falls, excluding puncture events like gunshot wounds and stabbings; (2) patients who underwent CT scans for a definitive diagnosis; (3) a time interval not exceeding a year between the event and the CT examination; (4) age >18 years old; (5) no prior diagnosis of osteoporosis or receipt of osteoporosis treatment. Patients meeting any of the following criteria were excluded: (1) presence of skeletal diseases associated with an increased risk of fractures (such as osteogenesis imperfecta, or malignant tumors); (2) CT scan not including the L1 vertebra; (3) history of rib fractures; and (4) absence of reconstructed images with a thickness of ≤ 1.5 mm. **Figure 1** shows a flowchart of the study design.

The eligible patients from January 1, 2017, to December 31, 2018, formed the retrospective cohort. To validate our findings and assess the predictive value of the BMD-based model, our researchers prospectively collected data continuously from eligible patients undergoing CT scans due to chest impact events at the radiology department (single center) from February 13 to June 2, 2023, forming a prospective cohort. Following their CT examinations, their thin-slice CT images were immediately processed by an AI algorithm to obtain BMD measurements and predict rib fracture patterns. Subsequently, the rib fracture patterns predicted by the BMD-based model will be compared with diagnostic reports provided by radiologist after a standard waiting period to validate the predictive value of the model. Detailed scan parameters are described in the **Supplementary Methods** of the online **Supplementary Material**.

### AI-based BMD measurements

A two-stage, deep-learning model was adopted. First, a three-dimensional UNet model was employed to complete vertebral segmentation, segmenting trabecular bone while excluding cortical bone; then, a DenseNet model was used to complete vBMD prediction (**Figure 1**) (23, 24).

The three-dimensional UNet model used in the first stage consisted of an encoder and a decoder network, with feature-fusion achieved through skip connections between the encoding and decoding modules. In the encoder network, two 3 × 3 × 3 convolutional layers and one 2 × 2 × 2 max pooling layer (stride = 2) were used for feature extraction, followed by downsampling. Four downsampling operations were performed to extract features of different dimensions. In the decoder network, a 2 × 2 × 2 upsampling convolutional layer and two 3 × 3 × 3 convolutional layers were used for feature extraction, followed by four upsampling operations. The final network layer adjusted the number of channels to the desired number of classes using a 1 × 1 convolutional layer.

In the second stage, the DenseNet model for BMD detection was divided into two parts: feature extraction and fully connected regression detection, with BMD measured using quantitative CT as the training target. The feature extraction part consisted of three Dienes blocks, each of which comprised an equal number of 1 × 1 × 1 and 3 × 3 × 3 convolutional layers. The fully connected regression detection head contained two layers of 2 × 2 × 2 global average pooling. The input size of the model was [16,32,32].

**Figure 2:**
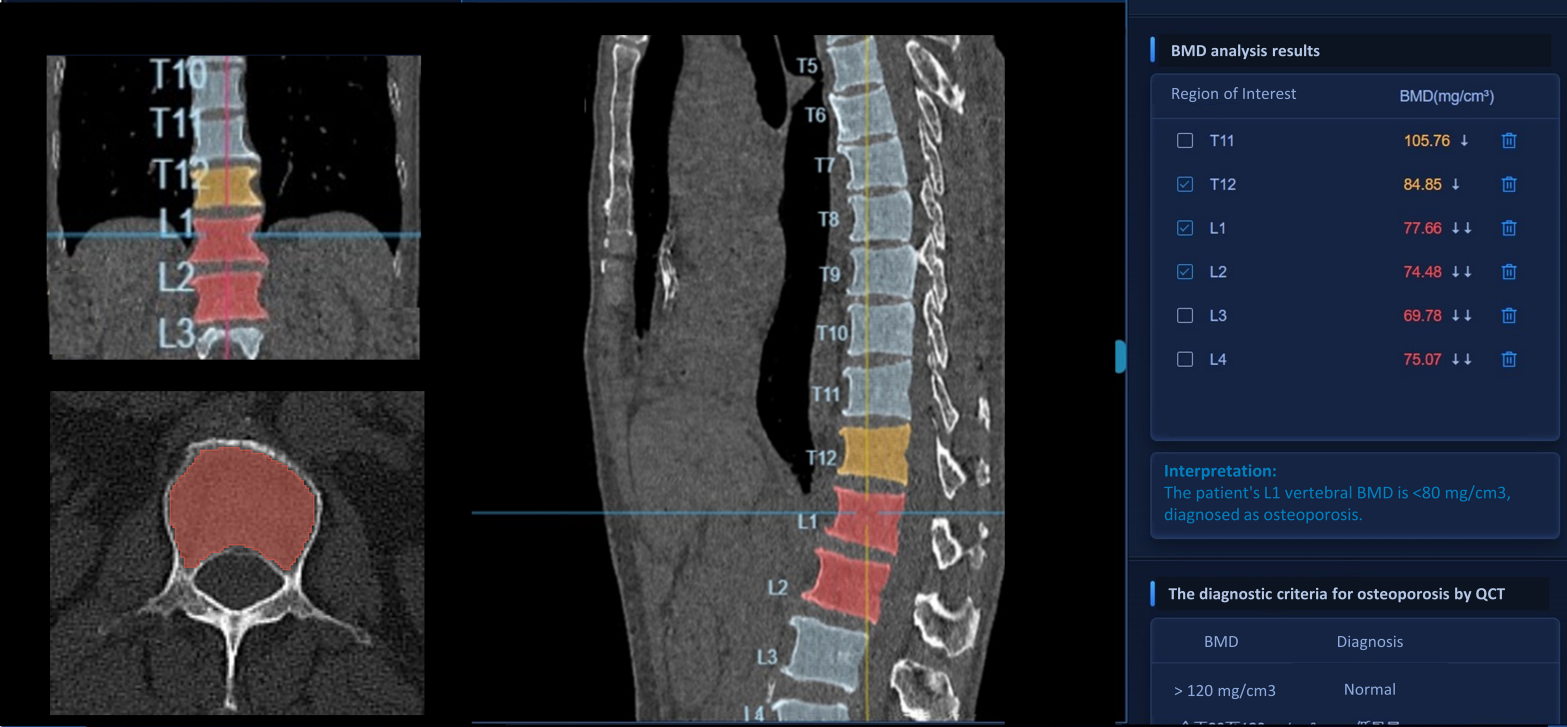
The detailed AI interface of BMD measurement. The left column of this interface comprises, from top to bottom, the patient’s coronal, axial, and three-dimensional reconstructed CT images, respectively. The middle column displays the patient’s sagittal CT images, while the right column presents the corresponding BMD results of the vertebrae. Vertebrae with BMD results <80 mg/m3 are annotated in red on the CT images, along with their respective BMD values. Vertebrae with BMD results in the range of 80-120 mg/m3 are marked in yellow on the CT images, together with their corresponding BMD values. BMD, bone mineral density; CT, computed tomography; AI, artificial intelligence.

We restricted our analysis to the L1 vertebra, previously considered as the optimal target for opportunistic BMD measurements using CT images (25–27). BMD status was categorized as normal (> 120 mg/cm^3^), osteopenic (80–120 mg/cm^3^), or osteoporotic (≤ 80 mg/cm^3^) based on the recommendation of the International Society for Clinical Densitometry and the American College of Radiology (28, 29). This criterion was considered applicable in the Chinese population (30). The AI interface of BMD measurement is displayed in **Figure 2**.

### Outcome measures

Our primary outcome was rib fracture patterns, confirmed by a radiologist (engaged in musculoskeletal imaging diagnosis for 15 years), through reference to both the CT diagnostic report and CT images, incorporating the following:

1. The presence of at least one rib fracture.
2. Characteristics: The number of fractured ribs and rib fracture sites; the location of the fractured rib(s) categorized as ribs 1–3, 4–7, and 8–12; and the presence of flail chest.
3. Classification based on fracture displacement: The degree of displacement of a single-rib fracture was classified as undisplaced, offset, or displaced (31). Patients were categorized into three groups: Group 1, undisplaced fractures only; Group 2, any offset but no displaced fractures; and Group 3, any case with displaced fractures (32). Patients who underwent internal fixation were excluded (as the fracture type present may change after internal fixation).

### Statistical analysis

Data were analyzed using R software version 4.3.0 (R Foundation for Statistical Computing, Vienna, Austria). Categorical data are given as frequencies and percentages. Medians and first to third quartiles (interquartile range) are used to summarize continuous variables. We compared variables between groups using Pearson’s χ^2^ or Fisher’s exact tests and Kruskal–Wallis tests for categorical and continuous variables, respectively. Furthermore, Bonferroni correction was used for *post-hoc* multiple comparisons. The association between BMD status and categorical outcomes was also assessed using binary logistic regression analysis. The impact of BMD status on the numbers of fractured ribs and rib fracture sites was assessed using negative binomial regression analysis. Unadjusted and adjusted (for sex and age [per 10 years]) logistic regression analyses were performed to calculate the odds ratios (ORs) and corresponding confidence intervals (CIs) to determine the association between BMD status and outcomes. Sensitivity analyses were performed using a three-knot piecewise polynomial model for age, with separate analyses for men and women, respectively. Statistical significance was set at P < 0.05.

We conducted further investigations into the ability of BMD alone and BMD combined with age and sex to predict rib fracture patterns using five-fold cross-validation. Model performance was evaluated using the area under the receiver operating characteristic curve (AUC), sensitivity, and specificity. Owing to the potential overestimation of performance when quantifying the predictive value of the model using derived data, we utilized a prospective cohort to calculate the performance metrics, enabling us to evaluate the performance after optimistic correction.

## Results

### Retrospective cohort

This cohort included 2,076 eligible patients (age: 55 [47–62] years; 777 [37.4%] females), of whom 954 (46%) had normal BMD, 806 (38.8%) had osteopenia, and 316 (15.2%) had osteoporosis. The median interval between CT scans and the occurrence of events was 125 days (IQR: 57-230, range: 0-354). Differences in the distribution of female patients (32.2% vs. 38.5% vs. 50.6%, P < 0.001) and age (age: 48 [40–55] vs. 58 [53–63] vs. 64 [58–70] years, P < 0.001) between the BMD groups were significant (**Supplementary Figure 1**). **Table 1** provides demographic characteristics and rib fracture features of the three BMD groups. Overall, 1,964 patients (94.6%) experienced single or multiple rib fractures, with a total of 10,886 fractured ribs and 14,091 fracture sites (number of fractured ribs: 5 [3–7]; number of fractured rib sites: 6 [3–9]) following chest trauma. Of the 2,076 patients, 335 (16.1%) were diagnosed with flail chest.

**Table 1:** Demographics and rib fracture features in the three BMD groups in the retrospective cohort.

Parameter	Overall	Normal BMD	Osteopenia	Osteoporosis	P value
Number of patients	2,076	954 (46.0%)	806 (38.8%)	316 (15.2%)	
Women	777 (37.4%)	307 (32.2%)	310 (38.5.%)	160 (50.6%)	< 0.001
Age (years), median (IQR)	55 (47–62)	48 (40–55)	58 (53–63)	64 (58–70)	< 0.001
>55 years old	979 (52.8%)	220 (23.1%)	489 (60.7%)	270 (85.4%)	< 0.001
Rib fracture	1,964 (94.6%)	884 (92.7%)	771 (95.7%)	309 (97.8%)	< 0.001
Number of ribs fractured, median (IQR)	5 (3–7)	4 (3–6)	5 (3–7)	6 (4–8)	< 0.001
Number of fracture sites on ribs, median (IQR)	6 (3–9)	5 (3–8)	6 (4–10)	8 (4–12)	< 0.001
Flail chest	335 (16.1%)	99 (10.4%)	154 (19.1%)	82 (25.9%)	< 0.001
Location of the fractured ribs					
1–3	1,073 (47.2%)	421 (44.1%)	448 (55.6%)	204 (64.6%)	< 0.001
4–7	1,782 (83.7%)	786 (82.3%)	703 (87.2%)	293 (92.7%)	< 0.001
8–12	1,057 (50.9%)	476 (49.9%)	424 (52.6%)	157 (49.7%)	0.47
Fracture type					
Group 1	1,146 (65.5%)	539 (69.8%)	437 (62.9%)	170 (60.3%)	0.016
Group 2	391 (22.4%)	151 (19.6%)	165 (23.7%)	75 (26.0%)	
Group 3	212 (12.1%)	82 (10.6%)	93 (13.4%)	37 (13.1%)	

IQR, interquartile range; BMD, bone mineral density.

### Association of BMD status with rib fractures

Osteoporosis and rib fractures were significantly associated in the unadjusted and sex- and age-adjusted analyses (OR: 3.5, 95% CI: 1.6–7.7 and OR: 2.7, 95% CI: 1.1–6.3, respectively).

**Figure 3:**
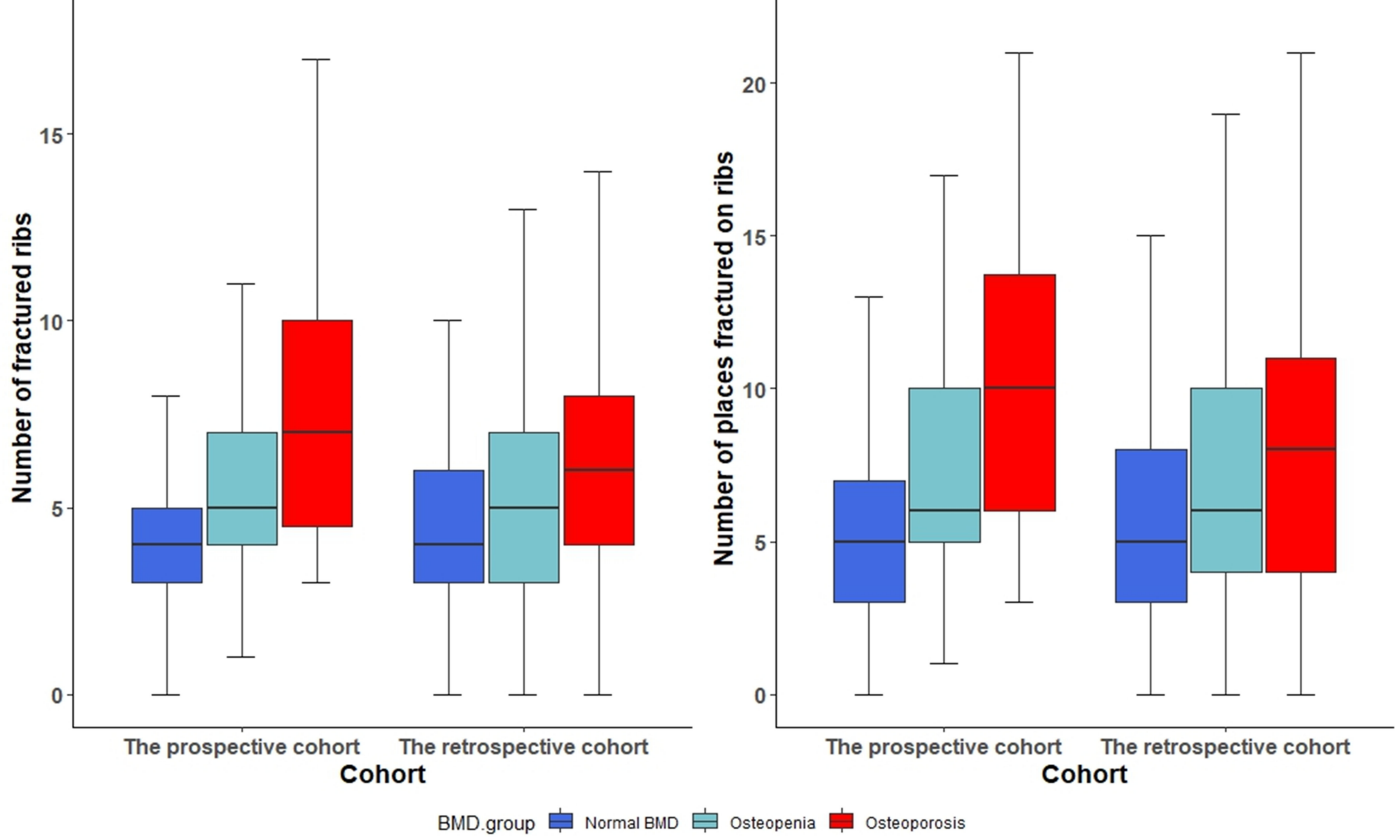
Distribution of number of fractured ribs and locations of rib fractures among bone mineral density groups. BMD, bone mineral density.

Moreover, BMD status was associated with the numbers of fractured ribs and rib fracture sites. As shown in **Figure 3**, the median numbers (IQR) of fractured ribs in the groups with normal BMD, osteopenia, and osteoporosis were 4 (IQR: 3–6), 5 (IQR: 3–7), and 6 (IQR:4–8), respectively. After Bonferroni correction, both the osteopenia and osteoporosis groups exhibited significantly higher numbers of fractured ribs compared with that in the normal BMD group (both P<0.001). The unadjusted negative binomial regression analysis results were OR: 1.2, 95% CI: 1.1–1.3 and OR: 1.4, 95% CI: 1.3–1.5 for the osteopenia and osteoporosis groups, respectively. Age- and sex-adjustment yielded similar results for the osteopenia and osteoporosis groups (OR: 1.2, 95% CI: 1.1–1.2 and OR: 1.3, 95% CI: 1.2–1.4, respectively). Furthermore, the median numbers (IQR) of fracture sites on ribs in the groups with normal BMD, osteopenia, and osteoporosis were 5(IQR: 3–8), 6 (IQR: 4–10), and 8 (IQR: 4–12), respectively. after Bonferroni correction, patients with both osteoporosis and osteopenia showed an increased number of fractured ribs compared with patients with a normal BMD (both P<0.001). The unadjusted negative binomial regression analysis results for the osteopenia and osteoporosis groups were OR: 1.3, 95% CI: 1.2–1.4 and OR: 1.5, 95% CI: 1.4–1.7, respectively. Sex- and age- adjustment did not significantly change the associations within the osteopenia (OR: 1.2, 95% CI: 1.1–1.3) or osteoporosis (OR: 1.3, 95% CI: 1.2–1.4) groups.

### Association of BMD status with flail chest

BMD status was significantly associated with flail chest. Among a total of 954 patients with normal BMD, 806 patients with osteopenia, and 316 patients with osteoporosis, there were 99 (10.4%), 154(19.1%), and 82 (25.9%) with flail chest, respectively. In the Bonferroni *post-hoc* analysis, both the osteopenia and osteoporosis groups had higher proportions of patients with flail chest than that in the normal BMD group (both P<0.001). The unadjusted and sex- and age-adjusted analyses results revealed an association between osteopenia and flail chest (OR: 2.0, 95% CI: 1.6–2.6 and OR: 1.6, 95% CI: 1.2–2.1, respectively). The corresponding values indicating the association of osteoporosis with flail chest were OR: 3.0, 95% CI: 2.2–4.2 and OR: 2.0, 95% CI: 1.4–3.0, respectively.

### Association of BMD status with rib fracture location

BMD status was associated with fractures in ribs 1–3. Overall, 1,073 of 2,076 (47.2%) patient fractures occurred in ribs 1–3 following chest trauma. In the Bonferroni *post-hoc* analysis, both osteopenia (449/806, 55.7%) and osteoporosis (204/316, 64.6%) were associated with higher proportions of fractures in ribs 1–3 compared with that for normal BMD (422/954, 44.2%) (both P<0.001). The unadjusted and sex- and age-adjusted analyses results revealed the association of osteopenia with fractures of ribs 1–3 (OR: 1.6, 95%: 1.3–1.9 and OR: 1.5, 95% CI: 1.2, 1.8, respectively); the corresponding values for osteoporosis were OR: 2.3, 95% CI: 1.8–3.0 and OR: 2.1, 95% CI: 1.5–2.8, respectively.

Overall, 1,782 of 2,076 (83.7%) patient fractures occurred in ribs 4–7 following chest trauma. In the Bonferroni *post-hoc* analysis, both osteopenia (703/806, 87.2%) and osteoporosis (294/316, 93.0%) were associated with higher proportions of fractures in ribs 4–7 than that for normal BMD (786/954, 82.4%) (both P < 0.001). The unadjusted and sex- and age-adjusted analyses revealed an association of osteoporosis with fractures of ribs 4–7 (OR: 2.7, 95% CI: 1.7–4.3 and OR: 2.2, 95% CI 1.4–3.6, respectively).

Overall, 1,057 of 2,076 (50.9%) patient fractures occurred in ribs 8–12 following chest trauma, with no significant associations.

**Supplementary Figure 2** illustrates the fracture frequency per rib in the normal and decreased BMD (osteopenia and osteoporosis) groups.

### Association of BMD status with fracture type

We excluded 217 patients (10.6%) due to the presence of internal fixation and subsequentially analyzed rib fracture types in the remaining 1,859 patients (89.4%). Of the 1,859 patients, 1,749 (94.1%) had at least one rib fracture. Group 1, Group 2, and Group 3 comprised 65.5% (n = 1146), 22.4% (n = 391), and 12.1% (n = 212), respectively. The fracture types for each BMD status are presented in **Table 1**. In the unadjusted ordinal multinomial analysis, osteopenia (OR: 1.4, 95% CI: 1.1–1.7) and osteoporosis (OR: 1.5, 95% CI: 1.1–1.9) contributed to the displacement of rib fracture ends. However, after sex and age adjustments, the associations between decreased BMD (osteopenia and osteoporosis) and fracture type were no longer significant.

**Figure 4:**
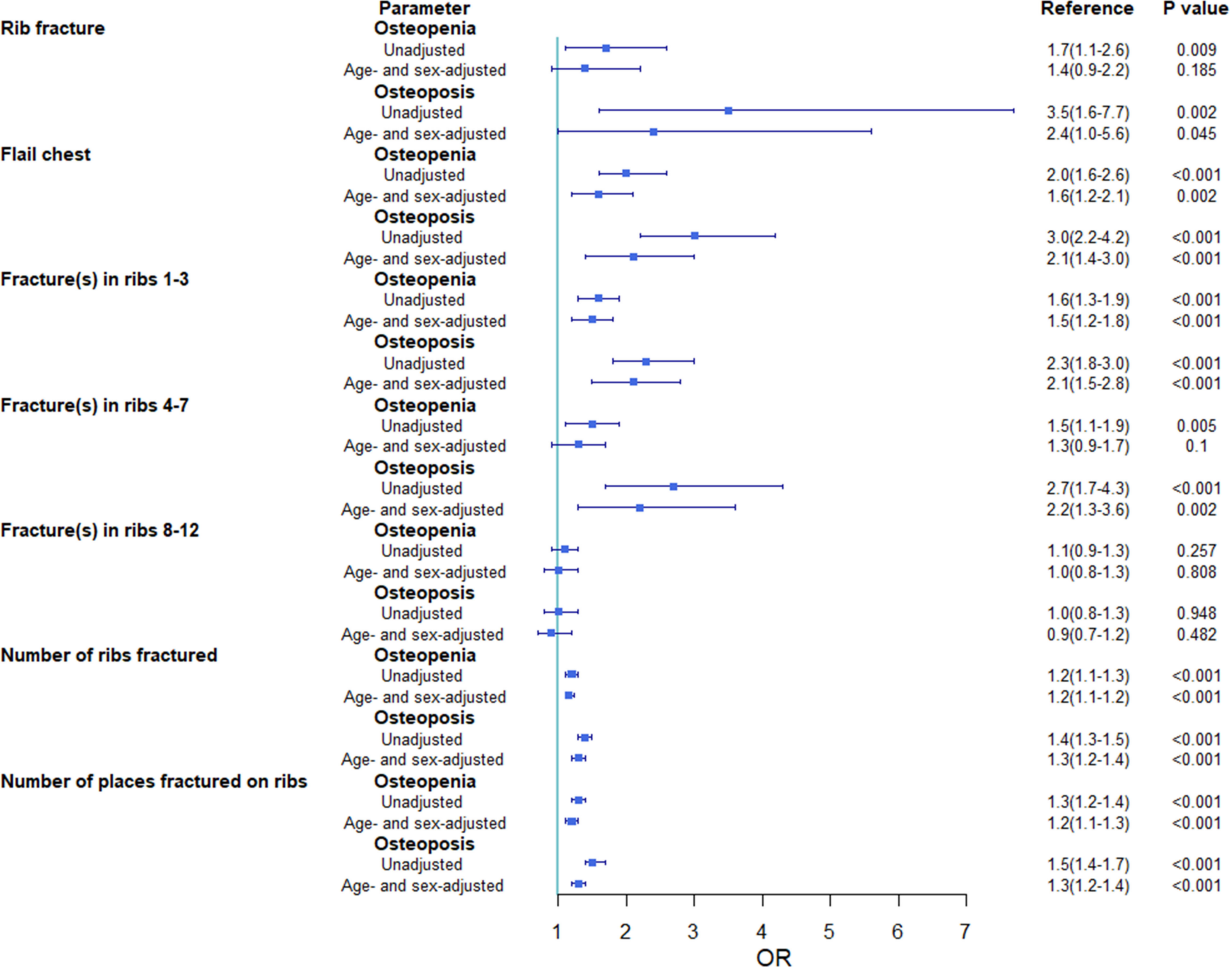
Unadjusted and adjusted odds ratios (95% confidence intervals) of events for the osteopenia and osteoporosis groups compared with that for the normal bone mineral density group. OR, odds ratio.

The outcomes according to BMD group are shown in **Figure 4** (**Supplementary Table 1**). Sensitivity analyses, including a three-knot piecewise polynomial model for age, separate analyses for men and women, and separate analyses for patients over 55 years old (**Supplementary Tables 2**-**4**), did not substantially change our results.

### Prospective cohort

The prospective cohort included 205 eligible patients (age [IQR], 55 [44–64] years; 90 [43.9%] women), of whom 92 (44.9%) had normal BMD, 74 (36.1%) had osteopenia, and 39 (19.0%) had osteoporosis. The median interval between CT scans and the occurrence of events was 92 days (IQR: 46.5-163.5, range: 0-304). The differences in sex distribution (32.6% vs. 50.0% vs. 59.0%, P = 0.009) and age (45 [37–55] vs. 58 [51–66] vs. 67 [60–71] years, P < 0.001) among the BMD groups were significant (**Supplementary Figure 1**). Conversely, differences in BMD status distribution and demographic characteristics did not differ significantly between the prospective and retrospective cohorts (**Table 2**). Fracture characteristics of the prospective cohort are described in the **Supplementary Results** and **Table 3**.

**Table 2:** Comparisons of bone mineral density status and demographic characteristics between retrospective and prospective cohorts.

	Retrospective cohort	Prospective cohort	P value
Women	777 (37.4%)	90 (43.9%)	0.680
Age (years), median (IQR)	55 (47–62)	55 (44–64)	0.939
BMD group			
Normal BMD	954 (46.0%)	92 (44.9%)	0.343
Osteopenia	806 (38.8%)	74 (36.1%)	
Osteoporosis	316 (15.2%)	39 (19.0%)	

BMD, bone mineral density; IQR, interquartile range.

**Table 3:** Demographics and rib fracture features of the three bone mineral density groups in the prospective cohort.

Parameter	Overall	Normal BMD	Osteopenia	Osteoporosis	P value
Number of patients	205	92 (44.9%)	74 (36.1%)	39 (19.0%)	
Women	90 (43.9%)	30 (32.6%)	37 (50.0%)	23 (59.0%)	0.009
Age (years), median (IQR)	55 (44–64)	45 (37–55)	58 (51–66)	67 (60–71)	<0.001
>55years old	94 (45.9%)	19 (20.7%)	41 (55.4%)	34 (87.2%)	<0.001
Rib fracture	200 (97.6%)	87 (94.6%)	74 (100.0%)	39 (100.0%)	0.082
Number of ribs fractured, median (IQR)	5 (4–7)	4 (3–6)	5 (4–7)	7 (4–10)	<0.001
Number of places fractured, median (IQR)	6 (4–10)	5 (3–7)	6 (5–10)	10 (6, 14)	<0.001
Flail chest	50 (24.4%)	13 (14.1%)	21 (28.4%)	16 (41.0%)	0.003
Location of fractured ribs					
1–3	132 (63.4%)	49 (53.3%)	47 (63.5%)	33 (92.3%)	<0.001
4–7	188 (91.7%)	77 (83.7%)	72 (97.3%)	39 (100.0%)	0.001
8–12	94 (45.9%)	40 (43.5%)	33 (44.6%)	21 (53.8%)	0.533
Fracture type					
Group 1	93 (50.8%)	43 (53.8%)	36 (53.7%)	14 (38.9%)	0.262
Group 2	53 (29.0%)	18 (22.5%)	21 (31.3%)	14 (38.9%)	
Group 3	37 (20.2%)	19 (23.8%)	10 (14.9%)	8 (22.2%)	
Event type					
Traffic accident (patients as passengers or drivers)	169 (82.4%)	74 (80.4%)	65 (87.8%)	30 (76.9%)	0.261
Traffic accident (patients as pedestrians)	20 (9.8%)	8 (8.7%)	6 (8.1%)	6 (15.4%)	
Falls	4 (2.0%)	1 (1.1%)	2 (2.7%)	1 (2.6%)	
Violent acts	12 (5.9%)	9 (9.8%)	1 (1.4%)	2 (5.1%)	

BMD, bone mineral density; IQR, interquartile range.

### Predictive value of BMD status for rib fracture patterns

#### Performance in the retrospective cohort

**Figure 5:**
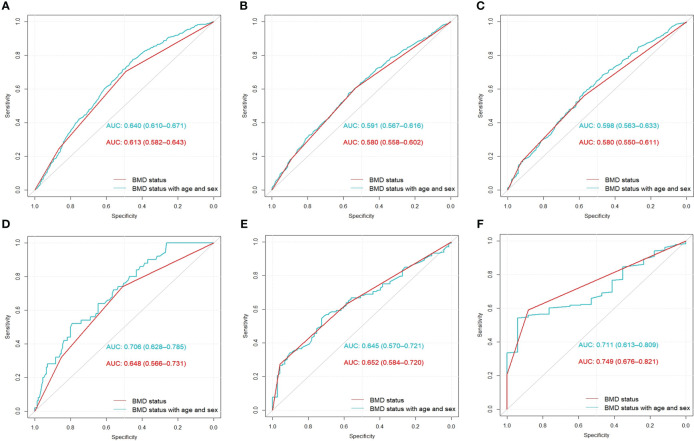
Area under the receiver operating characteristic curve results for the retrospective **(A–C)** and prospective **(D–F)** cohorts. The AUCs from left to right represent the predictions of flail chest, fractures in ribs 1–3, and fractures in ribs 4–7, respectively. AUC, area under the receiver operating characteristic curve; BMD, bone mass density.

Using BMD status only, the AUC values for predicting flail chest, fracture(s) in ribs 1–3, and fracture(s) in ribs 4–7 were 0.613 (95% CI: 0.582–0.643), 0.580 (95% CI: 0.558–0.602), and 0.580 (95% CI: 0.550–0.611), respectively. After incorporating BMD status, sex, and age into the prediction model, the corresponding AUC values were 0.640 (95% CI 0.610–0.671), 0.591 (95% CI: 0.567–0.616), and 0.598 (95% CI: 0.550–0.611), respectively (**Figure 5**).

#### Performance in the prospective cohort

Using the same parameters used in the retrospective cohort, the AUC values of using BMD status only for predicting flail chest, fracture(s) in ribs 1–3, and fracture(s) in ribs 4–7 were 0.648 (95% CI: 0.566–0.731), 0.652 (95% CI: 0.584–0.720), and 0.749 (95% CI: 0.676–0.821), respectively the corresponding AUC values of incorporating BMD status, sex, and age were 0.706 (95% CI: 0.628–0.785), 0.645 (95% CI: 0.570–0.721), and 0.711 (95% CI: 0.613–0.809), respectively (**Figure 5**). The detailed results are shown in **Table 4**.

**Table 4:** Receiver operating characteristic analysis results in the retrospective and prospective cohorts^a^.

		AUC	P value	SENS	SPEC
Flail chest					
BMD only	R-C	0.613 (0.582–0.643)	Ref	0.705 (0.651–0.752)	0.491 (0.468–0.515)
	P-C	0.648 (0.566–0.731)		0.740 (0.30–0.860)	0.529 (0.445–0.890)
BMD with age and sex	R-C	0.640 (0.610–0.671)	<0.001	0.770 (0.606–0.818)	0.466 (0.428–0.624)
	P-C	0.706 (0.628–0.785)	0.001	0.700 (0.440–1.000)	0.697 (0.377–0.897)
Fracture(s) in ribs 1–3					
BMD only	R-C	0.580 (0.558–0.602)	Ref	0.607 (0.578–0.634)	0.532 (0.503–0.560)
	P-C	0.652 (0.584–0.720)		0.326 (0.220–0.697)	0.932 (0.562–1.00)
BMD with age and sex	R-C	0.591 (0.567-0.616)	0.025	0.600 (0.361–0.771)	0.554 (0.376–0.799)
	P-C	0.645 (0.570–0.721)	0.683	0.557 (0.306–0.693)	0.753 (0.589–0.973)
Fracture(s) in ribs 4–7					
BMD only	R-C	0.579 (0.548–0.609)	Ref	0.559 (0.264–0.583)	0.579 (0.529–0.942)
	P-C	0.749 (0.676–0.821)		0.590 (0.516–0.660)	0.882 (0.706–1.000)
BMD with age and sex	R-C	0.598 (0.563–0.633)	0.049	0.611 (0.414–0.798)	0.553 (0.355–0.744)
	P-C	0.711 (0.613–0.809)	0.350	0.553 (0.452–0.638)	0.942 (0.824–1.000)

AUC, area under the receiver operating characteristic curve; SENS, sensitivity; SPEC, specificity; BMD, bone mineral density; R-C, retrospective cohort; P-C, prospective cohort; Ref, reference.

a 95% confidence intervals were obtained by setting the seed number to 123 and performing 1,000 bootstrap iterations.

We investigated the predictive value of BMD in a subset of patients aged over 55 who had experienced traffic accidents (either as passengers or drivers) for rib fracture patterns (**Supplementary Table 5**). The AUC values of using BMD status only for predicting flail chest, fracture(s) in ribs 1–3, and fracture(s) in ribs 4–7 were 0.629 (95% CI: 0.519–0.740), 0.685 (95% CI: 0.572–0.798), and 0.914 (95% CI: 0.874–0.954); The corresponding AUC values, combining BMD status, gender, and age, were 0.678 (95% CI: 0.560–0.795), 0.675 (95% CI: 0.551–0.799), and 0.889 (95% CI: 0.815–0.963).

## Discussion

We demonstrated that patients with chest trauma and decreased BMD had higher rib fracture rates and numbers of fracture sites, with a higher likelihood of fractures in ribs 1–3 and 4–7 as well as increased susceptibility to flail chest. Furthermore, we created a model encompassing BMD, sex, and age that moderately predicted rib fracture patterns.

Our results represent that rib fractures, even with trauma, are associated with decreased BMD. However, decreased BMD presents as a silent condition, with patients generally not undergoing BMD screening prior to fractures, leading to fracture treatment constituting a significant portion of expenses associated with osteoporosis (33). Opportunistic BMD assessment using CT-AI, conducted through CT imaging for other clinical purposes, may aid in identifying patients at an elevated risk of fractures and provide additional information regarding rib fracture patterns, intending to raise awareness in patients and enabling consideration of preventive and treatment.

Previous studies have extensively explored the association between BMD and fractures based on CT images (34–38). Chalhoub et al. (35) found that a greater risk of various fracture types was associated with low BMD. Other studies have investigated the predictive value of BMD for fractures using CT images (15, 39, 40); Gruenewald et al. (15) utilized the vBMD measurements of L1 to predict osteoporosis-related fractures and achieved satisfactory performance (AUC: 0.937, 95% CI: 0.867–0.977). Nevertheless, most previous studies have focused on traditional osteoporotic fractures (hip or spine), with limited exploration of the role of BMD in trauma. Prins et al. (41) investigated the association between BMD and rib fractures using DXA and showed that BMD impacted rib fractures. However, additional DXA scans increased the scan time and radiation exposure. Furthermore, this small-sample study (n = 119) did not establish an association between BMD and rib fracture patterns.

This study had some limitations First, it was conducted at a single center, the patient data were derived from individuals who underwent chest trauma evaluation at our institution. These patients had all experienced chest impact events and underwent CT scans for the purpose of a definitive diagnosis, often for forensic clinical assessment. This particular feature may have resulted in a limited number of patients without fractures, potentially rendering the sample insufficient to comprehensively represent the entire patient population. Second, our inability to obtain the actual accident circumstances made it impossible to differentiate between high- and low-energy fractures in trauma cases. Consequently, the correlation between BMD and low-energy fractures may have been underestimated due to the inclusion of high-energy fractures. However, non-osteoporotic fractures resulting from high-energy trauma are associated with low BMD (17). Future studies should include detailed accident information to provide improved correlation results. Finally, the model performance in predicting patterns by combining BMD status, age, and sex was moderate, indicating that factors other than these variables were influential. Future large-scale prospective studies should explore other risk factors and incorporate vBMD derived from CT-AI to assess fracture risk and patterns accurately.

Our study provides evidence for an association between BMD status and traumatic rib fractures. We further highlighted the potential predictive value of BMD status regarding the risk of rib fractures. Opportunistic BMD screening based on CT-AI can be utilized for the identification of individuals with decreased BMD, along with their fracture patterns.

## Data availability statement

The original contributions presented in the study are included in the article/**Supplementary Material**, further inquiries can be directed to the corresponding author/s.

## Ethics statement

The studies involving humans were approved by Ethics committee/institutional review board of Huadong hospital. The studies were conducted in accordance with the local legislation and institutional requirements. The ethics committee/institutional review board waived the requirement of written informed consent for participation from the participants or the participants’ legal guardians/next of kin because Because of the nature of retrospective study.

## Author contributions

YT: Data curation, Investigation, Methodology, Software, Writing – original draft. WH: Funding acquisition, Project administration, Resources, Supervision, Validation, Writing – review & editing. XX: Data curation, Formal analysis, Methodology. ML: Conceptualization, Funding acquisition, Investigation, Resources, Supervision, Validation, Visualization, Writing – review & editing. LJ: Conceptualization, Data curation, Funding acquisition, Investigation, Methodology, Project administration, Resources, Supervision, Validation, Visualization, Writing – original draft, Writing – review & editing.

## References

[1] Eghbalzadeh K Sabashnikov A Zeriouh M Choi YH Bunck AC Mader N. Blunt chest trauma: a clinical chameleon. Heart (2018) 104(9):719–24. doi: 10.1136/heartjnl-2017-312111 29203574

[2] Zhou J Wang T Belenkiy I Hardcastle TC Rouby JJ Jiang B. Management of severe trauma worldwide: implementation of trauma systems in emerging countries: China, Russia and South Africa. Crit Care (2021) 25(1):286. doi: 10.1186/s13054-021-03681-8 34372903 PMC8352140

[3] Schoell SL Weaver AA Vavalle NA Stitzel JD. Age- and sex-specific thorax finite element model development and simulation. Traffic Inj Prev (2015) 16(Suppl 1):S57–65. doi: 10.1080/15389588.2015.1005208 26027976

[4] Chrysou K Halat G Hoksch B Schmid RA Kocher GJ. Lessons from a large trauma center: impact of blunt chest trauma in polytrauma patients-still a relevant problem? Scand J Trauma Resusc Emerg Med (2017) 25(1):42. doi: 10.1186/s13049-017-0384-y 28427480 PMC5399315

[5] Statistics CNBo. Statistical bulletin on national economic and social development of the People's Republic of China in 2022 (2023) National Bureau of Statistics. Available at: https://www.stats.gov.cn/sj/zxfb/202302/t20230228_1919011.html (Accessed February 28, 2023).

[6] Brasel KJ Moore EE Albrecht RA deMoya M Schreiber M Karmy-Jones R. Western Trauma Association Critical Decisions in Trauma: Management of rib fractures. J Trauma Acute Care Surg (2017) 82(1):200–3. doi: 10.1097/ta.0000000000001301 27779590

[7] Jentzsch T Neuhaus V Seifert B Moos RM Simmen HP Schmitz CEW. Are the rib fracture score and different computed tomography measures of obesity predictors for mortality in patients with rib fractures? A retrospective cohort study. Eur J Trauma Emerg Surg (2022) 48(1):243–53. doi: 10.1007/s00068-020-01483-1 PMC8825425 32892237

[8] Shulzhenko NO Zens TJ Beems MV Jung HS O'Rourke AP Liepert AE. Number of rib fractures thresholds independently predict worse outcomes in older patients with blunt trauma. Surgery (2017) 161(4):1083–9. doi: 10.1016/j.surg.2016.10.018 27932031

[9] Hu J Zheng ZF Wang SH Si DL Yuan YQ Gao BL. Missed rib fractures on initial chest CT in trauma patients: time patterns, clinical and forensic significance. Eur Radiol (2021) 31(4):2332–9. doi: 10.1007/s00330-020-07310-w 33000304

[10] Talbot BS Gange CP Jr. Chaturvedi A Klionsky N Hobbs SK Chaturvedi A. Traumatic rib injury: patterns, imaging pitfalls, complications, and treatment. Radiographics (2017) 37(2):628–51. doi: 10.1148/rg.2017160100 28186860

[11] Dehghan N Mah JM Schemitsch EH Nauth A Vicente M McKee MD. Operative stabilization of flail chest injuries reduces mortality to that of stable chest wall injuries. J Orthop Trauma (2018) 32(1):15–21. doi: 10.1097/bot.0000000000000992 28902086

[12] Reid IR. A broader strategy for osteoporosis interventions. Nat Rev Endocrinol (2020) 16(6):333–9. doi: 10.1038/s41574-020-0339-7 32203407

[13] Coughlan T Dockery F. Osteoporosis and fracture risk in older people. Clin Med (Lond) (2014) 14(2):187–91. doi: 10.7861/clinmedicine.14-2-187 PMC4953292 24715132

[14] Harris K Zagar CA Lawrence KV. Osteoporosis: common questions and answers. Am Fam Physician (2023) 107(3):238–46. Available at: https://www.aafp.org/pubs/afp/issues/2023/0300/osteoporosis.html. 36920813

[15] Gruenewald LD Koch V Martin SS Yel I Eichler K Gruber-Rouh T. Diagnostic accuracy of quantitative dual-energy CT-based volumetric bone mineral density assessment for the prediction of osteoporosis-associated fractures. Eur Radiol (2022) 32(5):3076–84. doi: 10.1007/s00330-021-08323-9 PMC9038932 34713330

[16] Sanders KM Pasco JA Ugoni AM Nicholson GC Seeman E Martin TJ. The exclusion of high trauma fractures may underestimate the prevalence of bone fragility fractures in the community: the Geelong Osteoporosis Study. J Bone Miner Res (1998) 13(8):1337–42. doi: 10.1359/jbmr.1998.13.8.1337 9718203

[17] Mackey DC Lui LY Cawthon PM Bauer DC Nevitt MC Cauley JA. High-trauma fractures and low bone mineral density in older women and men. Jama (2007) 298(20):2381–8. doi: 10.1001/jama.298.20.2381 18042915

[18] Jäckle K Lüken S Roch PJ Klockner FS Reinhold M Meier MP. Effect of a contrast agent on bone mineral density measurement in the spine and hip using QCT-conversion factor recommendation. J Clin Med (2023) 12(4):1456. doi: 10.3390/jcm12041456 36835991 PMC9963832

[19] Pickhardt PJ Summers RM Garrett JW Krishnaraj A Agarwal S Dreyer KJ. Opportunistic screening: radiology scientific expert panel. Radiol (2023) 307(5):e222044. doi: 10.1148/radiol.222044 PMC10315516 37219444

[20] Peng T Zeng X Li Y Li M Pu B Zhi B. A study on whether deep learning models based on CT images for bone density classification and prediction can be used for opportunistic osteoporosis screening. Osteoporos Int (2023). doi: 10.1007/s00198-023-06900-w. [Online ahead of print] PMC10786975 37670164

[21] Yang J Liao M Wang Y Chen L He L Ji Y. Opportunistic osteoporosis screening using chest CT with artificial intelligence. Osteoporos Int (2022) 33(12):2547–61. doi: 10.1007/s00198-022-06491-y 35931902

[22] Pan Y Shi D Wang H Chen T Cui D Cheng X. Automatic opportunistic osteoporosis screening using low-dose chest computed tomography scans obtained for lung cancer screening. Eur Radiol (2020) 30(7):4107–16. doi: 10.1007/s00330-020-06679-y PMC7305250 32072260

[23] Tan Y Liu M Chen W Wang X Peng H Wang Y. DeepBranch: deep neural networks for branch point detection in biomedical images. IEEE Trans Med Imaging (2020) 39(4):1195–205. doi: 10.1109/tmi.2019.2945980 31603774

[24] Huang G Liu Z Pleiss G Maaten LV Weinberger KQ. Convolutional networks with dense connectivity. IEEE Trans Pattern Anal Mach Intell (2022) 44(12):8704–16. doi: 10.1109/tpami.2019.2918284 31135351

[25] Kang JW Park C Lee DE Yoo JH Kim M. Prediction of bone mineral density in CT using deep learning with explainability. Front Physiol (2022) 13:1061911. doi: 10.3389/fphys.2022.1061911 36703938 PMC9871249

[26] Jang S Graffy PM Ziemlewicz TJ Lee SJ Summers RM Pickhardt PJ. Opportunistic Osteoporosis Screening at Routine Abdominal and Thoracic CT: Normative L1 Trabecular Attenuation Values in More than 20 000 Adults. Radiol (2019) 291(2):360–7. doi: 10.1148/radiol.2019181648 PMC6492986 30912719

[27] Gerety EL Hopper MA Bearcroft PW. The reliability of measuring the density of the L1 vertebral body on CT imaging as a predictor of bone mineral density. Clin Radiol (2017) 72(2):177.e9–.e15. doi: 10.1016/j.crad.2016.09.022 28340962

[28] Engelke K Adams JE Armbrecht G Augat P Bogado CE Bouxsein ML. Clinical use of quantitative computed tomography and peripheral quantitative computed tomography in the management of osteoporosis in adults: the 2007 ISCD Official Positions. J Clin Densitom (2008) 11(1):123–62. doi: 10.1016/j.jocd.2007.12.010 18442757

[29] Radiology ACo. ACR–SPR–SSR practice parameter for the performance of musculoskeletal quantitative computed tomography (QCT). Revised 2023. American College of Radiology. Available at: https://www.acr.org/-/media/ACR/Files/Practice-Parameters/qct (Accessed August 15, 2023).

[30] Cheng X Zhao K Zha X Du X Li Y Chen S. Opportunistic screening using low-dose CT and the prevalence of osteoporosis in China: A nationwide, multicenter study. J Bone Miner Res (2021) 36(3):427–35. doi: 10.1002/jbmr.4187 PMC7988599 33145809

[31] Edwards JG Clarke P Pieracci FM Bemelman M Black EA Doben A. Taxonomy of multiple rib fractures: Results of the chest wall injury society international consensus survey. J Trauma Acute Care Surg (2020) 88(2):e40–e5. doi: 10.1097/ta.0000000000002282 31590175

[32] Clarke PTM Simpson RB Dorman JR Hunt WJ Edwards JG. Determining the clinical significance of the Chest Wall Injury Society taxonomy for multiple rib fractures. J Trauma Acute Care Surg (2019) 87(6):1282–8. doi: 10.1097/ta.0000000000002519 31688826

[33] Aibar-Almazán A Voltes-Martínez A Castellote-Caballero Y Afanador-Restrepo DF Carcelén-Fraile MDC López-Ruiz E. Current status of the diagnosis and management of osteoporosis. Int J Mol Sci (2022) 23(16):9465. doi: 10.3390/ijms23169465 36012730 PMC9408932

[34] Lee SJ Graffy PM Zea RD Ziemlewicz TJ Pickhardt PJ. Future osteoporotic fracture risk related to lumbar vertebral trabecular attenuation measured at routine body CT. J Bone Miner Res (2018) 33(5):860–7. doi: 10.1002/jbmr.3383 PMC5935538 29314261

[35] Lee SJ Anderson PA Pickhardt PJ. Predicting future hip fractures on routine abdominal CT using opportunistic osteoporosis screening measures: A matched case-control study. AJR Am J Roentgenol (2017) 209(2):395–402. doi: 10.2214/ajr.17.17820 28570093

[36] Adams AL Fischer H Kopperdahl DL Lee DC Black DM Bouxsein ML. Osteoporosis and hip fracture risk from routine computed tomography scans: the fracture, osteoporosis, and CT utilization study (FOCUS). J Bone Miner Res (2018) 33(7):1291–301. doi: 10.1002/jbmr.3423 PMC6155990 29665068

[37] Liu Y Yu A Li K Wang L Huang P Geng J. Differences in spine volumetric bone mineral density between grade 1 vertebral fracture and non-fractured participants in the China action on spine and hip status study. Front Endocrinol (Lausanne) (2022) 13:1013597. doi: 10.3389/fendo.2022.1013597 36387886 PMC9647629

[38] Chalhoub D Orwoll ES Cawthon PM Ensrud KE Boudreau R Greenspan S. Areal and volumetric bone mineral density and risk of multiple types of fracture in older men. Bone (2016) 92:100–6. doi: 10.1016/j.bone.2016.08.014 PMC5056840 27554426

[39] Gruenewald LD Koch V Yel I Eichler K Gruber-Rouh T Alizadeh LS. Association of phantomless dual-energy CT-based volumetric bone mineral density with the prevalence of acute insufficiency fractures of the spine. Acad Radiol (2022) 30(10):2110–7. doi: 10.1016/j.acra.2022.11.020 36577605

[40] Dieckmeyer M Löffler MT El Husseini M Sekuboyina A Menze B Sollmann N. Level-specific volumetric BMD threshold values for the prediction of incident vertebral fractures using opportunistic QCT: A case-control study. Front Endocrinol (Lausanne) (2022) 13:882163. doi: 10.3389/fendo.2022.882163 35669688 PMC9165054

[41] Prins JTH Van Lieshout EMM Reijnders MRL Verhofstad MHJ Wijffels MME. Rib fractures after blunt thoracic trauma in patients with normal versus diminished bone mineral density: a retrospective cohort study. Osteoporos Int (2020) 31(2):225–31. doi: 10.1007/s00198-019-05219-9 PMC7010612 31828365

