# Supplementary material for: Traumatic rib fracture patterns associated with bone mineral density statuses derived from CT images
Source: Front Endocrinol (Lausanne). 2023 Dec 12;14:1304219. doi: 10.3389/fendo.2023.1304219 (PMC10754511; doi:10.3389/fendo.2023.1304219)
Supplement: Supplementary file 1 [file DataSheet_1.docx]

**Online Supplemental Materials**

**Supplemental Methods.** Computed Tomography Image Acquisition.

**Supplemental Figure 1.** Distribution of Sex and Age among Bone Mineral Density Groups in the Retrospective and Prospective Cohorts

**Supplemental Figure 2.** Distribution of Number of Fractured Ribs and Rib Fractures among the Bone Mineral Density Groups in the Retrospective Cohort

**Supplemental Table 1.** Unadjusted and Adjusted Odds Ratios (95% Confidence Intervals) for Events in the Bone Mineral Density Groups

**Supplemental Table 2.** Regression Analyses with Splines

**Supplemental Table 3.** Sensitivity Analyses for Sex

**Supplemental Results.** Detailed Fracture Characteristics of the Prospective Cohort

**Supplemental Table 4.** Sensitivity Analyses including patients aged over 55 only

This supplemental material was provided by the authors to provide readers with additional information on their work.

**Supplemental Methods**

**Computed Tomography Image Acquisition**

Computed tomography (CT) images were acquired using three CT scanners: a 16-cm coverage detector CT scanner (Revolution CT, GE Healthcare, Chicago, IL, USA) and two dual-source CT scanners (Somatom Definition Flash and Somatom Definition Drive, Siemens Healthcare, Erlangen, Germany). The following parameters were used: 100–120 kVp, depending on the patient’s body size; 100–200 mAs; pitch, 0.75–1.5; and collimation, 1–1.25 mm. All imaging data were reconstructed into 512 × 512 matrices using either a bone or a medium-sharpness reconstruction algorithm with a slice thickness of 1–1.5 mm. The scanning range included all the ribs.

**Supplemental Figure 1. Distribution of Sex and Age Among Bone Mineral Density Groups in the Retrospective and Prospective Cohorts**


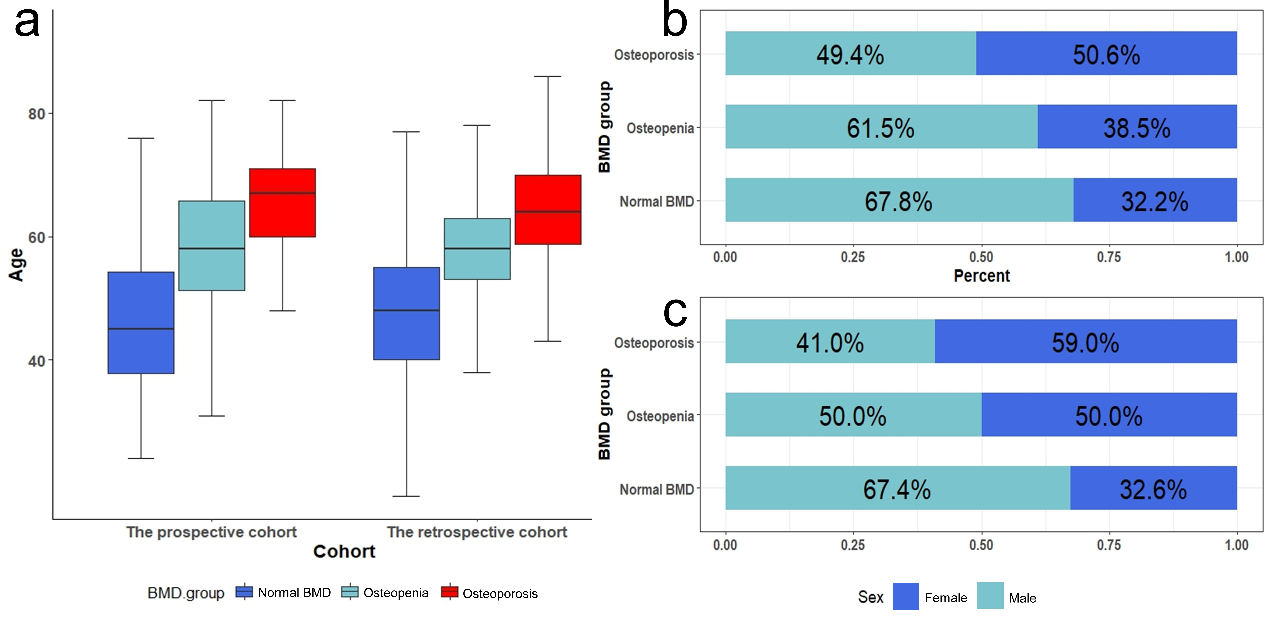


(a) Age distributions in the retrospective and prospective cohorts. Sex distributions in the (b) retrospective and (c) prospective cohorts. Abbreviations: BMD, bone mineral density

**Supplemental Figure 2. Distribution of Number of Fractured Ribs and Rib Fractures Among Bone Mineral Density Groups in the Retrospective Cohort**


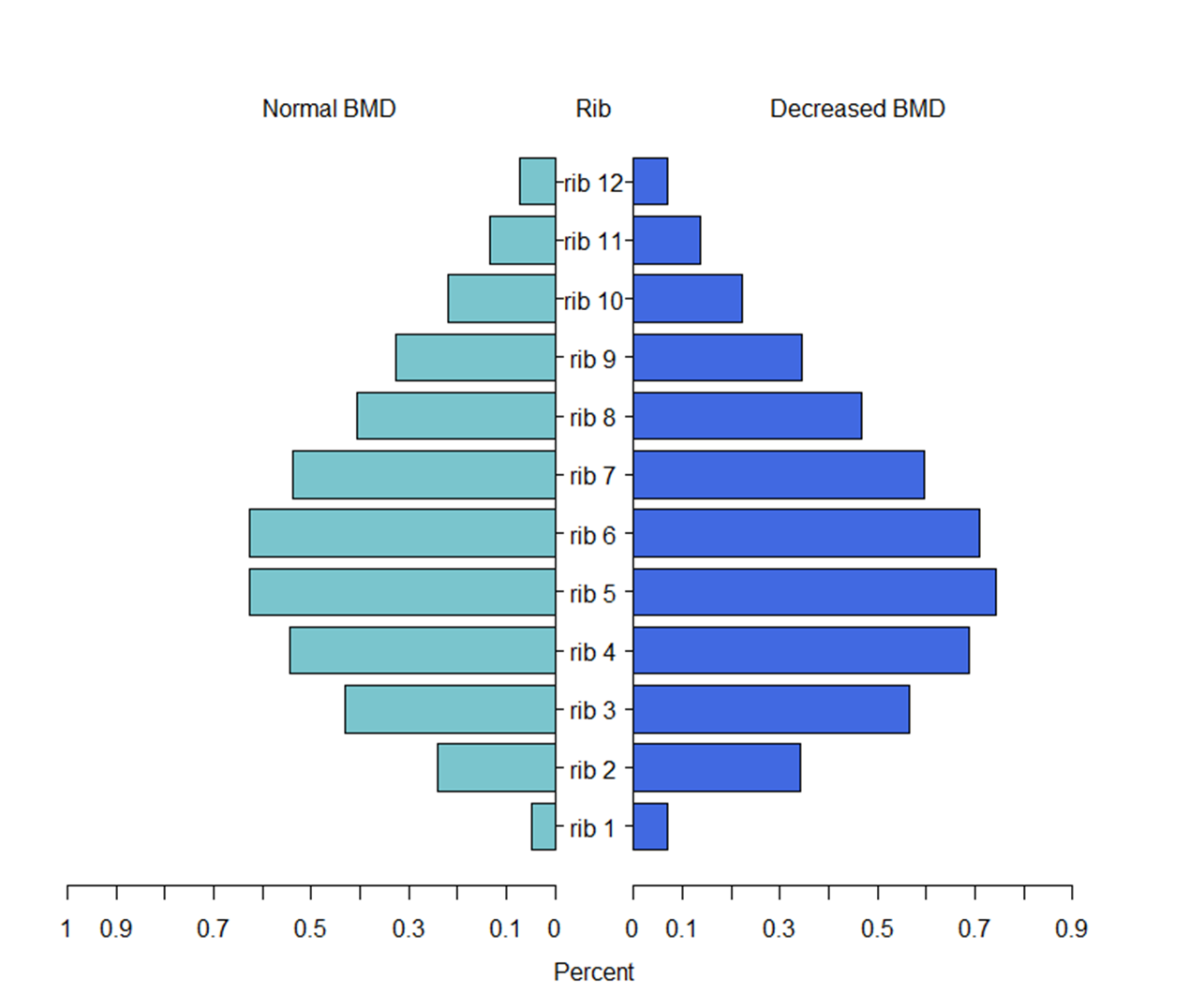


Abbreviations: BMD, bone mineral density

**Supplemental Table 1 Unadjusted and Adjusted Odds Ratios (95% Confidence Intervals) for Events in the Bone Mineral Density Groups**

| **Parameter** | **Overall** | **Normal BMD** | **Osteopenia** | **Osteoporosis** |
| --- | --- | --- | --- | --- |
| Number of patients | 2,076 | 954 (44.9%) | 806 (37.9%) | 316 (14.8%) |
| Rib fracture | 1,964 (94.6%) | 884 (92.7%) | 771 (95.7%) | 309 (97.8%) |
| Unadjusted OR | NA | Ref | 1.7 (1.1–2.6) [0.009] ^a^ | 3.5 (1.6–7.7) [0.002] |
| Age- and sex-adjusted OR | NA | Ref | 1.4 (0.9–2.2) [0.185] | 2.4 (1.0–5.6) [0.045] |
| Flail chest |  |  |  |  |
| Unadjusted OR | NA | Ref | 2.0 (1.6–2.6) [<0.001] | 3.0 (2.2–4.2) [<0.001] |
| Age- and sex-adjusted OR | NA | Ref | 1.6 (1.2–2.1) [0.002] | 2.1 (1.4–3.0) [<0.001] |
| Fracture(s) in ribs 1–3 |  |  |  |  |
| Unadjusted OR | NA | Ref | 1.6 (1.3–1.9) [<0.001] | 2.3 (1.8–3.0) [<0.001] |
| Age- and sex-adjusted OR | NA | Ref | 1.5 (1.2–1.8) [<0.001] | 2.1 (1.5–2.8) [<0.001] |
| Fracture(s) in ribs 4–7 |  |  |  |  |
| Unadjusted OR | NA | Ref | 1.5 (1.1–1.9) [0.005] | 2.7 (1.7–4.3) [<0.001] |
| Age- and sex-adjusted OR | NA | Ref | 1.3 (0.9–1.7) [0.1] | 2.2 (1.3–3.6) [0.002] |
| Fracture(s) in ribs 8–12 |  |  |  |  |
| Unadjusted OR | NA | Ref | 1.1 (0.9–1.3) [0.257] | 1.0 (0.8–1.3) [0.948] |
| Age- and sex-adjusted OR | NA | Ref | 1.0 (0.8–1.3) [0.808] | 0.9 (0.7–1.2) [0.482] |
| Fracture type |  |  |  |  |
| Unadjusted OR | NA | Ref | 1.4 (1.1–1.7) [<0.001] | 1.5 (1.1–1.9) [<0.001] |
| Age- and sex-adjusted OR | NA | Ref | 1.0 (0.8–1.3) [0.843] | 0.9 (0.7–1.3) [0.668] |
| Number of ribs fractured |  |  |  |  |
| Unadjusted OR | NA | Ref | 1.2 (1.1–1.3) [<0.001] | 1.4 (1.3–1.5) [<0.001] |
| Age- and sex-adjusted OR | NA | Ref | 1.2 (1.1–1.2) [<0.001] | 1.3 (1.2–1.4) [<0.001] |
| Number of places fractured on ribs |  |  |  |  |
| Unadjusted OR | NA | Ref | 1.3 (1.2–1.4) [<0.001] | 1.5 (1.4–1.7) [<0.001] |
| Age- and sex-adjusted OR | NA | Ref | 1.2 (1.1–1.3) [<0.001] | 1.3 (1.2–1.4) [<0.001] |

Abbreviations: BMD, bone mineral density; NA, not applicable; Ref, reference; OR, odds ratio.

^a^ Data in parentheses are 95% confidence intervals and data in brackets are P values.

**Supplemental Table2. Regression Analyses with Splines**

|  | **Rib fracture** | **Flail chest** | **Fracture(s) in ribs 1–3** | **Fracture(s) in ribs 4–7** |
| --- | --- | --- | --- | --- |
| Variable | Sex- and age-adjusted odds ratios | | |  |
| BMD group |  |  |  |  |
| Normal | Ref | Ref | Ref | Ref |
| Osteopenia | 1.4 (0.9–2.2) | 1.5 (1.1–2.1) ^a^ | 1.5 (1.2–1.8) ^a^ | 1.3 (1.0–1.7) |
| Osteoporosis | 2.6 (1.1–6.2) ^a^ | 2.1 (1.4–3.0) ^a^ | 2.1 (1.5–2.8) ^a^ | 2.4 (1.4–4.0) ^a^ |
| Sex | 1.0 (0.7–1.5) | 1.1 (0.9–15) | 0.8 (0.7–1.0) ^a^ | 0.9 (0.7–1.2) |
| Age (spline)^b^ | NA | NA | NA | NA |

Abbreviations: BMD, bone mineral density; Ref, reference; NA, not available.

^a^ Statistically significant values (P < 0.05).

^b^ Odds ratios based on spline regression with a three-knot piecewise polynomial model for age.

**Supplemental Table 3. Sensitivity Analyses by Sex**

1. **Sensitivity analyses including women only**

|  | **Rib fracture** | | **Fracture(s) in ribs 1–3** | | **Fracture(s) in ribs 4–7** | | **Flail chest** | | **Number of ribs fractured** | | **Number of places fractured on ribs** | |
| --- | --- | --- | --- | --- | --- | --- | --- | --- | --- | --- | --- | --- |
| Variable | Unadjusted OR | Age-adjusted OR | Unadjusted OR | Age-adjusted OR | Unadjusted OR | Age-adjusted OR | Unadjusted OR | Age-adjusted OR | Unadjusted OR | Age-adjusted OR | Unadjusted OR | Age-adjusted OR |
| BMD groups |  |  |  |  |  |  |  |  |  |  |  |  |
| Normal | Ref | Ref | Ref | Ref | Ref | Ref | Ref | Ref | Ref | Ref | Ref | Ref |
| Osteopenia | 1.7 (0.8–3.4) | 1.1 (0.5–2.5) | 1.5 (1.1–2.0) ^b^ | 1.4 (1.0–2.0) | 1.3 (0.8–2.1) | 1.1 (0.6–1.8) | 1.5 (0.9–2.4) | 1.3 (0.7–2.1) | 1.2 (1.1 -1.3) ^a^ | 1.1 (1.0-1.2) ^a^ | 1.3 (1.1 -1.4) ^a^ | 1.1 (1.0-1.3) |
| Osteoporosis | 2.3 (0.8–6.2) | 1.2 (0.4–4.0) | 2.3 (1.5–3.4) ^b^ | 2.0 (1.3–3.3) ^b^ | 2.2 (1.1–4.4) ^b^ | 1.7 (0.8–3.6) | 3.1 (1.8–4.9) ^b^ | 2.2 (1.2–4.2) ^b^ | 1.4 (1.3-1.6) ^a^ | 1.3 (1.1-1.4) ^a^ | 1.7 (1.5-1.9) ^a^ | 1.4 (1.2-1.6) ^a^ |

1. **Sensitivity analyses including men only**

|  | **Rib fracture** | | **Fracture(s) in ribs 1-3** | | **Fracture(s) in ribs 4-7** | | **Flail chest** | | **Number of ribs fractured** | | **Number of places fractured on ribs** | |
| --- | --- | --- | --- | --- | --- | --- | --- | --- | --- | --- | --- | --- |
| Variable | Unadjusted OR | Age-adjusted OR | Unadjusted OR | Age-adjusted OR | Unadjusted OR | Age-adjusted OR | Unadjusted OR | Age-adjusted OR | Unadjusted OR | Age-adjusted OR | Unadjusted OR | Age-adjusted OR |
| BMD groups |  |  |  |  |  |  |  |  |  |  |  |  |
| Normal | Ref | Ref | Ref | Ref | Ref | Ref | Ref | Ref | Ref | Ref | Ref | Ref |
| Osteopenia | 1.7 (1.1–3.0) ^a^ | 1.5 (0.8–2.6) | 1.6 (1.3–2.0) ^a^ | 1.6 (1.2–2.2) ^a^ | 1.5 (1.1–2.1) ^a^ | 1.4 (1.0–2.0) ^a^ | 2.4 (1.7–3.4) ^a^ | 1.8 (1.3–2.6) ^a^ | 1.3 (1.2-1.3) ^a^ | 1.2 (1.1-1.3) ^a^ | 1.4 (1.3-1.5) ^a^ | 1.2 (1.1-1.4) ^a^ |
| Osteoporosis | 6.3 (1.5–26.2) ^a^ | 4.7 (1.1–20.5) ^a^ | 2.2 (1.5–3.1) ^a^ | 2.0 (1.4–3.0) ^a^ | 3.0 (1.6–5.8) ^a^ | 2.6 (1.3–5.1) ^a^ | 3.0 (1.9–4.7) ^a^ | 1.9 (1.2–3.1) ^a^ | 1.3 (1.2-1.5) ^a^ | 1.2 (1.1-1.4) ^a^ | 1.5 (1.3-1.7) ^a^ | 1.3 (1.1-1.5) ^a^ |

Abbreviations: BMD, bone mineral density; OR, odds ratio; Ref, reference.

^a^ Statistically significant values (P < 0.05).

**Supplemental Table 4. Sensitivity Analyses including patients aged over 55 only**

|  | **Rib fracture** | | **Fracture(s) in ribs 1–3** | | **Fracture(s) in ribs 4–7** | | **Flail chest** | | **Number of ribs fractured** | | **Number of places fractured on ribs** | |
| --- | --- | --- | --- | --- | --- | --- | --- | --- | --- | --- | --- | --- |
| Variable | Unadjusted OR | Age-adjusted OR | Unadjusted OR | Age-adjusted OR | Unadjusted OR | Age-adjusted OR | Unadjusted OR | Age-adjusted OR | Unadjusted OR | Age-adjusted OR | Unadjusted OR | Age-adjusted OR |
| BMD groups |  |  |  |  |  |  |  |  |  |  |  |  |
| Normal | Ref | Ref | Ref | Ref | Ref | Ref | Ref | Ref | Ref | Ref | Ref | Ref |
| Osteopenia | 1.0 (0.5-2.2) | 1.0 (0.5-2.2) | 1.5(1.1-2.0) ^a^ | 1.5(1.1-2.0) ^a^ | 1.4(0.8,2.0) | 1.3(0.8-2.0) | 1.3 (0.8-2.0) | 1.3 (0.9-2.0) | 1.1(1.0-1.2) | 1.1(1.0-1.2) | 1.2 (1.0-1.3) ^a^ | 1.1 (1.0-1.3) ^a^ |
| Osteoporosis | 2.1 (0.7-5.9) | 2.2 (0.7-6.3) | 2.1(1.4-3.0) ^a^ | 2.1(1.5-3.1) ^a^ | 2.4(1.3-4.4) ^a^ | 2.5(1.3-4.6) ^a^ | 1.7 (1.1-2.7) ^a^ | 1.7 (1.1-2.7) ^a^ | 1.3(1.1-1.4) ^a^ | 1.2 (1.1-1.4) ^a^ | 1.4 (1.2-1.5) ^a^ | 1.3 (1.2-1.5) ^a^ |

Abbreviations: BMD, bone mineral density; OR, odds ratio; Ref, reference.

^a^ Statistically significant values (P < 0.05).

**Supplemental Results**

**Detailed Fracture Characteristics of the Prospective Cohort**

Rib fractures occurred following chest trauma in 200 of 205 (97.6%) patients. The incidence of rib fractures following chest trauma was higher in both the osteopenia (74/74, 100.0%) and osteoporosis (39/39, 100.0%) groups than that in the normal BMD group (87/92, 94.6%). For the entire prospective cohort, the total and median (IQR) numbers of fractured ribs were 1,180 and 5 (IQR: 4–7), respectively, and the total and median (IQR) numbers of rib fractures were 1,566 and 6 (IQR: 4–10), respectively. After Bonferroni correction, both osteopenia (median [IQR], 5 [4–7]) and osteoporosis (median [IQR], 7 [4–10]) groups had significantly higher numbers of fractured ribs than that in the normal BMD group (median [IQR], 4 [3–6]). Regarding the number of places fractured on the ribs, both osteopenia (median [IQR], 6 [5–10]) and osteoporosis (median [IQR], 10 [6–14]) groups had more sites fractured on the ribs than that in the normal BMD group (median [IQR], 5 [3–7]) after Bonferroni correction. These results are consistent with those for the retrospective cohort.

Fractures occurred in ribs 1–3 after chest trauma in 132 of 205 (64.4%) patients. Compared with that in the normal BMD group (49/32, 44.9%), both the osteopenia (47/74, 63.5%) and osteoporosis (36/39, 92.3%) groups had higher proportions of fractures in ribs 1–3. Fractures occurred in ribs 4–7 following chest trauma in 188 of 205 (91.7%) patients. Compared with that in the normal BMD group (77/92, 83.7%), both the osteopenia (72/74, 97.3%) and osteoporosis (39/39, 100.0%) groups had higher proportions of fractures in ribs 4–7. Fractures occurred in ribs 8–12 following chest trauma in 94 of 205 (45.9%) patients. The proportions did not differ among the three groups. These results are consistent with those observed for the retrospective cohort.

50 of 205 (24.4%) patients developed flail chest. After excluding patients (17/200, 8.5%) who underwent surgical internal fixation treatment, an analysis was performed on the remaining 183 patients to assess the fracture types. Group 1, Group 2, and Group 3 comprised 50.8% (n=93), 29.0% (n=53), and 20.2% (n=37), respectively. No significant difference was observed in the distribution of fracture types among the three BMD groups (Table 2). Information regarding the demographics and rib fracture features for each BMD status are detailed in Table 3.

**Supplemental Table 5. Receiver operating characteristic analysis in** **the subset of patients aged over 55 who experienced traffic accidents in the prospective cohorts**

|  | **AUC** | **P value** | **SENS** | **SPEC** |
| --- | --- | --- | --- | --- |
| **Flail chest** | | | | |
| BMD only | 0.629 (0.519–0.740) | Ref | 0.613 (0.410–0.968) | 0.661(0.311–0.810) |
| BMD with age and sex | 0.678 (0560–0.795) | 0.0867 | 0.810 (0.722–0.940) | 0.689 (0.556–0.789) |
| **Fracture(s) in ribs 1–3** | | | | |
| BMD only | 0.685 (0.572-0.780) | Ref | 0.455 (0.355–0.881) | 0.870 (0.351–1) |
| BMD with age and sex | 0.675 (0.551-0.780) | 0.678 | 0.552 (0.313–0.940) | 0.826 (0.350–1) |
| **Fracture(s) in ribs 4–7** | | | | |
| BMD only | 0.914 (0.874–0.954) | Ref | 0.828 (0.747–0.908) | 1 (1–1) |
| BMD with age and sex | 0.862 (0.782–0.931) | 0.371 | 0.862 (0.782–0.931) | 1 (1–1)) |

Abbreviations: AUC, area under the receiver operating characteristic curve; SENS, sensitivity; SPEC, specificity; BMD, bone mineral density; R-C, retrospective cohort; P-C, prospective cohort; Ref, reference.

^a^ 95% confidence intervals were obtained by setting the seed number to 123 and performing 1,000 bootstrap iterations.
